# Dr. Patrick Blaikie-Smith

**Published:** 1909-04-03

**Authors:** 


The death has occurred at San Remo of Dr. Patrick Blaikie-Smith, who practised till recently at Aberdeen. He was educated at Aberdeen University and Middlesex Hospital, and was consulting physician to the Aberdeen Royal Infirmary and physician to the Invalid Ladies' Home, San Remo. He formerly held the post of assistant professor of chemistry and examiner in medicine at the University of Aberdeen, and that of physician and lecturer on clinical medicine at Aberdeen Royal Infirmary.

